# Evaluation of the Effect of a Gamma Irradiated DBM-Pluronic F127 Composite on Bone Regeneration in Wistar Rat

**DOI:** 10.1371/journal.pone.0125110

**Published:** 2015-04-21

**Authors:** Tamer Al Kayal, Daniele Panetta, Barbara Canciani, Paola Losi, Maria Tripodi, Silvia Burchielli, Priscilla Ottoni, Piero Antonio Salvadori, Giorgio Soldani

**Affiliations:** 1 Institute of Clinical Physiology- CNR, Pisa, Italy; 2 University & IRCCS AOU San Martino—IST, National Institute for Cancer Research, DIMES, Genova, Italy; 3 CNR/Tuscany “G. Monasterio” Foundation, Pisa Italy; University of Connecticut Health Center, UNITED STATES

## Abstract

Demineralized bone matrix (DBM) is widely used for bone regeneration. Since DBM is prepared in powder form its handling properties are not optimal and limit the clinical use of this material. Various synthetic and biological carriers have been used to enhance the DBM handling. In this study we evaluated the effect of gamma irradiation on the physical-chemical properties of Pluronic and on bone morphogenetic proteins (BMPs) amount in DBM samples. In vivo studies were carried out to investigate the effect on bone regeneration of a gamma irradiated DBM-Pluronic F127 (DBM-PF127) composite implanted in the femur of rats. Gamma irradiation effects (25 kGy) on physical-chemical properties of Pluronic F127 were investigated by rheological and infrared analysis. The BMP-2/BMP-7 amount after DBM irradiation was evaluated by ELISA. Bone regeneration capacity of DBM-PF127 containing 40% (w/w) of DBM was investigated in transcortical holes created in the femoral diaphysis of Wistar rat. Bone porosity, repaired bone volume and tissue organization were evaluated at 15, 30 and 90 days by Micro-CT and histological analysis. The results showed that gamma irradiation did not induce significant modification on physical-chemical properties of Pluronic, while a decrease in BMP-2/BMP-7 amount was evidenced in sterilized DBM. Micro-CT and histological evaluation at day 15 post-implantation revealed an interconnected trabeculae network in medullar cavity and cellular infiltration and vascularization of DBM-PF127 residue. In contrast a large rate of not connected trabeculae was observed in Pluronic filled and unfilled defects. At 30 and 90 days the DBM-PF127 samples shown comparable results in term of density and thickness of the new formed tissue respect to unfilled defect. In conclusion a gamma irradiated DBM-PF127 composite, although it may have undergone a significant decrease in the concentration of BMPs, was able to maintains bone regeneration capability.

## Introduction

Bone tissue has an excellent ability of regeneration in healthy individuals. However, when the defect exceeds a critical size or in pathological conditions (e.g. osteoporosis, tumors, cysts), impaired bone formation can occur. To date, the most effective graft materials for bone repair is the patient's own bone graft (autograft), due to its natural properties, such as optimal osteointegration, osteoconduction, and osteoinduction.

The use of autologous bone graft prevents implant rejection and disease transmission, but its disadvantages such as extensive surgery, limited graft quantity, donor site pain and morbidity has led to the development of graft substitutes for bone healing [1,2]. Excellent substitute requires being porous, osteoinductive, osteoconductive with a resorption rate that allows the bone healing process at the implanted site and a mechanical strength that maintains bone functionality.

Demineralized bone matrix (DBM), obtained by removing the mineral component from bone tissue with preservation of proteins content including bone morphogenetic proteins (BMPs), represents a valid alternative to the autograft use [3,4]. However, difficulties with handling and its tendency to migrate from graft site, are clinical problems associated with the use of powdered or particulate forms of DBM [5].

Various carrier materials, both natural and synthetic, including glycerol, Poloxamers, fibrin, hyaluronic acid, acellular matrix, gelatin, chitosan and PLGA [6–12], have been used to enhance the handling of DBM powder. There are several commercial products based on DBM combined with biocompatible and biodegradable materials, such as Grafton and Osteofil that showed osteoinductive properties in vitro and in vivo tests [13]. These DBM-based products in different conformations (e.g. injectable, sheet, strip, pastes) remain the preferred grafting material in bone regeneration and repair procedures.

Between the various carriers, Pluronic F127, a compound belonging to the category of poloxamers, appears to be an interesting carrier for DBM since it is a thermoreversible hydrogel that undergoes a sol-gel transition at body temperature above a critical gelation concentration, being, moreover, of utmost interest in optimizing drug formulation [14–17]. Pluronic F127 as thermogelling vehicle has the good properties of local inertia and prolonged drug residence [18]. In particular inhalation, oral solution, ophthalmic, topical and injectable preparations have been fully developed and present the advantages of promoting stabilization and water dissolution of many drugs. FDA guide has presented Poloxamer 407 as an inactive ingredient for different types of drugs [19].

An important requirement of sterilization procedure for all implantable medical devices based on DBM is to guarantee bioactivity of the products. The commonly used procedures for sterilization of medical device are steam [20], radiation [21] and ethylene oxide [22] that have previously demonstrated to induce partial or complete reduction in DBM endogenous biological activity.

Sterilization with gamma irradiation offers several advantages (simple, efficient, terminal treatment and convenient) that make it attractive in a growing number of situations. Several research papers described sterilization by radiation, which include a large variety of disposable medical products, sutures and implants, pharmaceuticals, cosmetics, and biological tissues [23].

Unfortunately, gamma irradiation can induce degradation effect by chain scission of synthetic and natural polymers usually employed as carriers [24,25]. Feasibility of using gamma irradiation as a sterilization procedure is therefore necessary when each compound for changes in biological activity or for induction of harmful products is applied.

There are conflicting results available about the effect of gamma irradiation on DBM bioactivity [26,27] and few studies on the effects of gamma irradiation on Pluronic [28], both taken separately, but there is a complete lack of information about the effect of gamma irradiation on composites made by DBM and Pluronic F127. Therefore, in this study we evaluated the effect produced by gamma irradiation on the physical-chemical properties of Pluronic and on BMPs amount released from irradiated and not irradiated DBM samples.

Furthermore, in vivo studies were carried out to investigate the effect on bone regeneration of a gamma irradiated DBM-Pluronic F127 (DBM-PF127) composite used as a filler of femoral defects produced in a young Wistar rat model. The bone healing effects were compared with unfilled (control) and Pluronic F127 filled defects.

## Materials and Methods

### Demineralized bone matrix preparation

Human bone tissue was supplied from TissueLab S.r.l (Naples, Italy). Briefly, soft tissue was removed and processed with biological detergents, and peroxide designed to cleanse and reduce lipid and marrow levels. Bones were demineralized by incubation in 0.6 M HCl for 3 h at room temperature. The decalcified bone was neutralized in phosphate solution, and lyophilized.

### DBM-PF127 composite preparation

A 20% (w/v) solution of Pluronic F127 in saline solution was prepared by using a magnetic stirrer at 4°C overnight. The Pluronic F127 powder was purchased from Sigma-Aldrich, MO, USA.

The DBM-PF127 composite was prepared mixing DBM particles (< 80 μm) and the Pluronic F127 solution at 4°C to obtain a composite containing 40% (w/w) DBM. The composite preparation has a form of putty at the room temperature.

### Gamma irradiation

Thirty aliquots taken from each sample (Pluronic F127 solution, DBM and DBM-PF127) were sealed in a nitrogen atmosphere and irradiated by gamma rays at -80°C from a Cobalt-60 chamber with a dose rate 6.4 kGy h^-1^ for 4 h to obtain 25 kGy final dose (Gammarad Italia Spa, Bologna, Italy). Aliquots from not irradiated preparations of Pluronic F127 and DBM were used as a control in rheological and chemical analysis, and BMP-2/BMP-7 quantification, respectively.

### Rheological analysis of Pluronic F127 solution

Rheological analyses were performed by a stress control rheometer (AR2000, T.A. Instruments Ltd., USA) equipped with a cone-plate geometry (diameter 60 mm, angle: 2 DEG and truncation 52 micron), automatic gap, operating in oscillation mode.

All samples were analyzed using a temperature sweep test, performed by stressing the samples at constant frequency (6.283 rad/sec), pressure (10 Pa) and temperature increments from 5 to 45°C at a rate of 1°C/min. Before each test, samples were left to equilibrate in the rheometer geometry at 5°C for 5 min. Each sample was analyzed in triplicate. Using this test it was possible to evaluate the variation of the sol-gel transition temperature after gamma irradiation.

### Chemical analysis of Pluronic F127 solution

Irradiated and not irradiated Pluronic F127 solutions were lyophilized before chemical analysis. Attenuated Total Reflectance Fourier Transform Infrared Spectroscopy (ATR-FTIR) was used to analyze changes in the chemical properties of Pluronic F127 following exposure to gamma radiation. Analysis was conducted using a Perkin Elmer Spectrum 65 equipped with ATR unit. Spectra were recorded between 650 and 4000 cm^-1^ and the principal absorption peaks were assigned at 2891 cm^-1^ (C-H stretch aliphatic), 1343 cm^-1^ (in-plane O-H bend) and 1111 cm^-1^ (C-O stretch).

### BMP-2 and BMP-7 quantification procedure in DBM

The bone morphogenetic proteins BMP-2 and BMP-7 were extracted from DBM with the guanidine HCl/EDTA method in accordance to a previously described protocol [29]. DBM samples (200 mg each) from four donors (named S1, S2, S3 and S4) were incubated for 7 days with 2 mL of extraction solution constituted of 4M Guanidin-HCl; 50 mM Tris; 50 mM EDTA, pH 7.4; 5 mM benzamidine HCl; 1 mM phenylmethyl-sulfonyl fluoride; and 0.1M ε-aminocaproic acid (all materials obtained from Sigma-Aldrich). After incubation the samples were dialyzed (cutoff of 3.5 kDa) in distilled water at 4°C for 24 h; the water was changed three times. After dialyzation the samples were centrifuged at 600g and the supernatant was stored at -80°C until the assay of the growth factors. Concentrations of BMP-2 and BMP-7 in irradiated and not irradiated DBM samples were determined according to manufacturer’s instructions using commercially available ELISA kits (R&D Systems, Inc., Minneapolis, MN, USA). BMPs concentrations were measured in triplicate in all samples. Statistical analysis was performed using StatView 5.0 (SAS Institute, Cary, NC, USA) by Student's t test. A value of p<0.05 was considered statistically significant.

### Sterility test procedure

The sterilized samples were immersed in a Mueller-Hinton broth for cultivation of microorganisms (Oxoid S.p.A., Milano, Italy) and maintained under agitation at 37°C for 5 days. Sterile broth was used as negative control, while unsterilized graft was used as positive control. Clouding of the broth indicates contamination and inefficient sterilization, while a clear and uncontaminated broth indicates efficient sterilization.

### Surgical procedure and sample harvesting

Thirty-six male Wistar rats, approximately 300 g in weight, age 3 months were purchased from Harlan Laboratories S.r.l. (Udine, Italy) at the age of 8 weeks. The rats were acclimatized to laboratory conditions for 10 days before surgery.

All animals received human care according to Italian Legislative Decree 116/92. This study was carried out in strict accordance with the recommendations regarding the use of animals for research purposes of the Italian Ministry of Health, Department of Veterinary, Public Health and Food Safety, which approved this protocol (Permit Number: 250/2011-B).

Rats were anesthetized by intraperitoneal injection of Zoletil (40 mg/kg) and Xylazine (5 mg/kg) and all efforts were made to minimize suffering. The side face of each hind limb was shaved and disinfected with Betadine and Neoxidina. After skin incision the muscles were opened out to highlight the lateral face of the femoral diaphysis. Then, transcortical holes (ø 3mm) were drilled bilaterally in the medial femoral diaphysis. The animals were randomly assigned to two experimental groups of 18 rats each: 1) DBM-PF127 composite filled and unfilled (control) defect in the femur and its contralateral, respectively; 2) Pluronic F127 and unfilled (control) defect in the femur and its contralateral, respectively. After suturing the surgical site and after the first CT scan as described in the following section, the animals were returned to their cages and fed with normal diet. Six rats of each group were euthanized by isoflurane inhalation 15 (early-term), 30 (medium-term) and 90 (long-term) days after implantation. The femurs were harvested and fixated in 10% buffered formalin for micro-CT and histological analysis.

### Micro-CT instrumentation and imaging protocols

#### Micro-CT scanner

The Xalt scanner (X-ray AnimaL Tomograph) was used for both in vivo and ex vivo imaging protocols [30]. This scanner allows to select the geometric magnification in the range 1.2–2.6 in order to optimize spatial resolution and field-of-view (FoV) depending on imaging protocol needs. For a single tomographic scan, the maximum transaxial FoV size is 80 mm in diameter for the ‘Large FoV’ (LF) setup, with an isotropic voxel size of 40 μm; the minimum voxel size is 18.4 μm in the ‘High Resolution’ (HR) setup. The minimum acquisition time is 43 s for fast, low-dose in vivo scans, and can be as high as 4 hours for ex vivo, low-contrast high resolution studies. Typical scan times are 2–10 min for in vivo imaging of mice and rats at 40–80 μm resolution, and 5–60 min for ex vivo scans of organs and biopsies at 18–40 μm resolution.

#### In vivo imaging protocol

**Fig 1:**
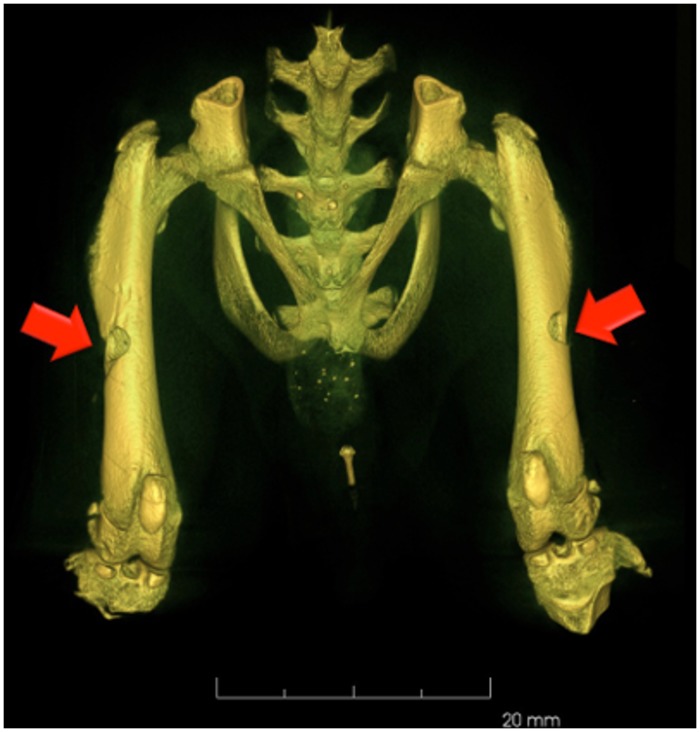
In vivo micro-CT image acquired after the surgical procedure showing the size and extent of the lesions.

In vivo longitudinal micro-CT scan was performed on all animals starting from the same day of the surgical procedure, right after the lesion induction (Fig 1). Scans were performed at 15 days (early-term group), 30 days (medium-term group) and 60 days (long-term group) to monitor relative lesion changes following different treatments. Due to the presence of radiosensitive organs in the Field of Measurement (FOM), such as the bladder and the gonads, we kept the cumulative absorbed dose over the whole follow-up period as low as possible by performing the in vivo scans using a 3 min protocol, 50 kV, 1 mm Al filtration, reconstructed at the isotropic resolution of 80 μm. All scans were done under general anesthesia with intraperitoneal injection of Zoletil and Xilazine and isofluorane inhalation for all the duration of the scan. In order to avoid motion artifacts, care was taken to ensure proper immobilization of the legs and pelvis during the acquisition time.

#### Ex vivo imaging protocol

After in vivo micro-CT scan and prior to histology, an ex vivo scan was performed with the HR setup (6 min total scan time, 50 kV, 1 mm Al filtration, reconstructed with the isotropic voxel size of 18.4 μm) on each explanted femur placed in a test tube and immersed in formalin. An ad hoc holder was realized in order to place the test tube at the tip of the animal bed, so as to avoid any other attenuating material in the micro-CT FOV. Using this setup, volumetric images of 700x700x1300 voxels were generated and then analyzed for the quantification of the bone regeneration.

#### Micro-CT image analysis

**Fig 2:**
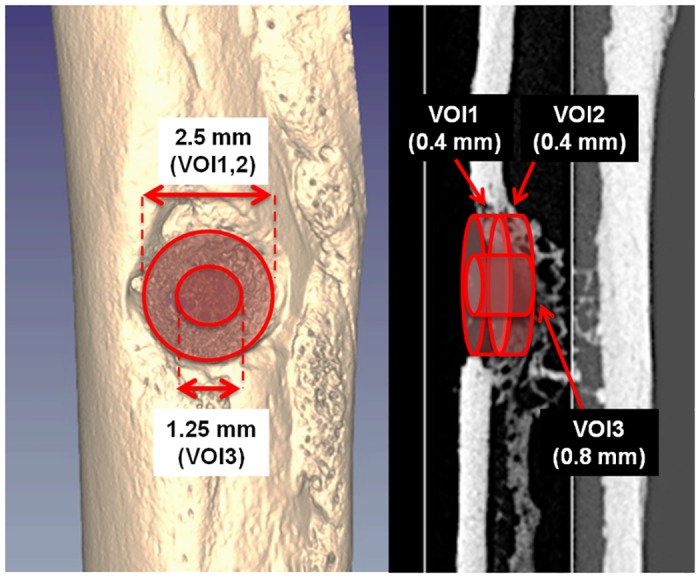
Representative ex vivo micro-CT images showing the VOIs used for quantitative analysis.

For each ex vivo scan, three cylindrical volumes of interest (VOI) centered on the were selected (Fig 2). The first VOI (VOI-1) has a diameter of 2,5 mm and a thickness of 0.4 mm, and was placed in such a way that a fully repaired femur would fill it completely with new bone. The second VOI (VOI-2) has the same size of VOI-1, but it was shifted towards the medullary canal by a distance equal to the its thickness (i.e., 0.4 mm). VOI-2 was used to take into account all those situations where the bone callus is shifted towards the medullary canal with respect to the normal condition, in such a way that the VOI-1 would be left almost empty even though the hole was completely closed by new bone. The third VOI (VOI-3) has a smaller diameter (1.25 mm) and a greater thickness (0.8 mm), and was used only to measure the average cortical thickness at the center of the lesion after 30 days and 90 days post-surgery. All the VOIs had a diameter that was intentionally smaller than the drill tip. This was necessary to exclude the margins of the lesions that could have increased the variance of our results, due to the low reproducibility of the drilling position and the strong variability of the undamaged bone thickness throughout the accessible positions and angles of the femur.

The analysis was performed using the BoneJ plugin [31] of the ImageJ software [32]. For each femur, the bone volume (BV), bone surface (BS) and surface/volume ratio (BS/BV) were measured in VOI-1 and VOI-2, whereas, the average cortical thickness (Ct.Th) was measured in VOI-3 For the Ct.Th analysis, a cutoff thickness of 20 μm has been selected to exclude the contribution of the trabeculae in the endosteal layer.

#### Statistical analysis

Quantitative micro-CT data were analyzed by comparing groups with unmatched two-tails t-test using Microsoft Excel 2011. Differences between groups were considered significant for p<0.05.

### Histology methods

The femurs were embedded in the light-curing resin Technovit 7200VLC (Kulzer, Bio-Optica, Milano, Italy), infiltrated for 21 days under vacuum, changing the resin every 7 days. Samples were polymerized by the EXAKT 520 polymerizator system. Curing was performed at 450 nm light with the temperature of the specimens never exceeding 40°C. The specimens were then prepared to be cut, according to the precision paralleling-guide procedure protocol, using the precision presses Exakt 401 and 402 Vacuum Adhesive Press. Sections were then cut using the EXAKT 310 CP cutting unit. The sections obtained were approximately 200 μm in thickness. Sections were then grinded to 20–30 μm using the EXAKT 400 CS micro grinding unit. EXAKT equipment was provided by EXAKT Advanced Technologies, Bio-Optica Milano, Italy. Sections were stained with Stevenel’s /Van Gieson (SVG) and Von Kossa stain. Briefly, SVG stain is a sequence of Stevenel’s blue stain prepared by mixing methylene blue (1%) (Sigma-Aldrich) and potassium permanganate (1.5%) (Carlo Erba, Milano, Italy) dissolved in boiling distilled water. Van Gieson picro-fuschin stain is prepared by first dissolving 0.1 g acid fuchsin (Sigma-Aldrich) in 10 ml of distilled water, followed by the addition of 100 ml of saturated picric acid (Nova Chimica Panreac, Milan, Italy). The colors in the samples depict mineralized bone in fuchsia; bone marrow, osteoid matrix and cellular cytoplasm in grades of blue, fibrous tissue in pink. Von Kossa stain is the result after immersion in 5% silver nitrate solution (Sigma-Aldrich) and exposure of the samples directly in sun light for 2 hours with counterstain of the nuclei in 0.1% Kernechtrot red solution 30 minutes at 37°C. The black color outlines the mineralized tissue while, nuclei are stained in red. Images were taken using Axiovert 200M microscope (Zeiss, Germany).

Other femur specimens were incubated in 1:100 v/v decalcified solution (Bio-Optica) for 5 h. After washing with deionized water for 90 min, the samples were dehydrated in increasing concentrations of ethanol (70 to 100%). They were embedded in paraffin and sectioned at 7 μm, fixed on poly-L-lysine coated glass slides, and stained with hematoxylin eosin.

## Results

### Rheological analysis

**Fig 3:**
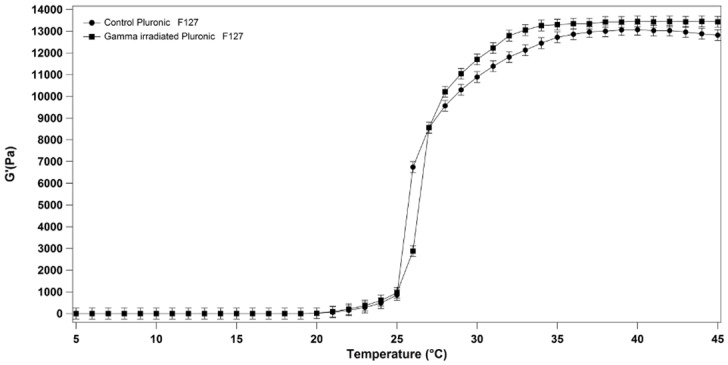
Storage modulus G’ of 20% (w/v) Pluronic F127 solution as a function of temperature (5°C to 45°C) for not irradiated and gamma irradiated samples.

No significant change in the sol-gel transition temperature was observed between a 20% (w/v) Pluronic F127 solution gamma irradiated with a dose of 25 kGy and not irradiated samples (Fig 3). In particular, the sol-gel transition temperature remained unchanged at 25°C for both irradiated and not irradiated solutions. However we have observed a change in the G’ value of gamma irradiated Pluronic F127 respect the control at temperature > 30°C.

### Chemical analysis

**Fig 4:**
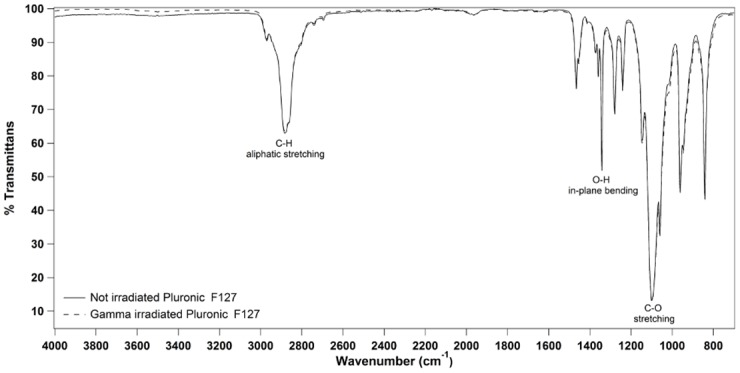
Representative ATR-FTIR spectra for the not irradiated and gamma irradiated Pluronic F127 samples.

No significant changes in the IR spectra of gamma irradiated and not irradiated 20% (w/v) Pluronic F127 solutions were observed on analyzed spectral regions from 650 cm^-1^ to 3500 cm^-1^. In particular, no effect on the principal functional groups, such as 2891 cm^-1^ (C-H stretch aliphatic), 1343 cm^-1^ (in-plane O-H bend) and 1111 cm^-1^ (C-O stretch), was observed following exposure to gamma irradiation (Fig 4).

### BMP-2 and BMP-7 concentration in DBM

**Fig 5:**
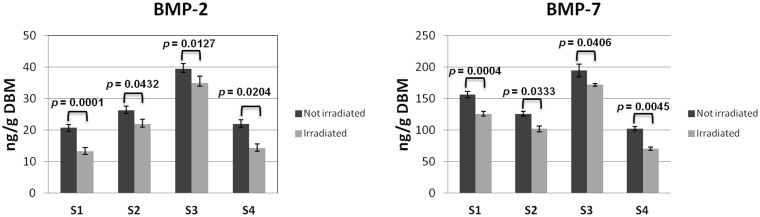
Concentration of BMP-2 (left) and BMP-7 (right) in gamma irradiated and not irradiated samples (*p<0.05).

The growth factors concentration in gamma irradiated and not irradiated DBM samples was determined by a commercial ELISA-kits. Fig 5 shows the growth factor amount correlated to DBM weight. In general, for each samples BMP-7>BMP-2 and the concentration of both decreases after gamma irradiation. The BMP-2 and BMP-7 percentage reduction in irradiated samples was 22% and 21% respectively in comparison to control. In all samples the decreasing of BMPs concentration in irradiated samples was statistically significant (p<0.05) in comparison to the relative not irradiated control.

### Sterility testing of DBM-PF127 composite

Following gamma irradiation with a dose of 25 kGy, all composite samples were rendered sterile. Only the not irradiated composite, used as positive control, determined the clouding of the Mueller—Hinton broth in the sterility test, indicating that a contamination of microorganisms was present.

### General animal health

No untoward animal deaths were observed in the experimental groups. None of the animals evidenced obvious morbidity. All animals gained weight during the study period and no animal exhibited infection or inflammation at the surgical site.

### Micro-CT imaging

The quantitative micro-CT analysis of bone lesions filled with DBM-PF127 composite evidenced the formation of trabecular bone 15 days after implantation, with subsequent formation of a dense trabecular network and a thick cortical region at 30 and 90 days, respectively, comparable with unfilled defect.

**Fig 6:**
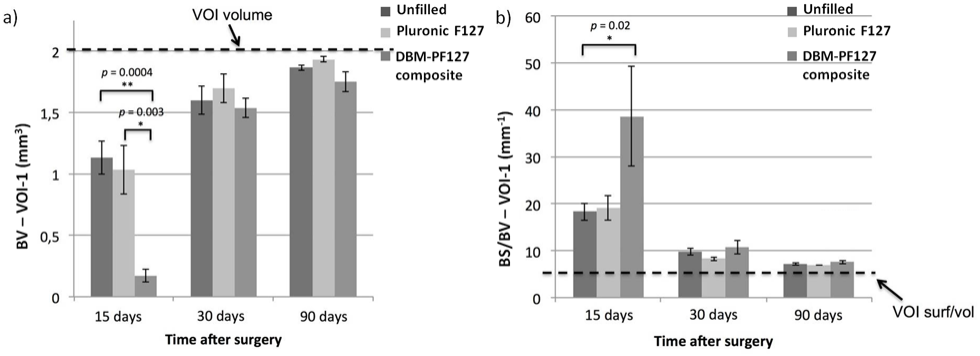
Quantitative results of bone repair as obtained with ex vivo micro-CT in VOI-1. In (**a**), the dashed line represents the total volume in the VOI-1 (2 mm^3^) which is the limit for 100% repair, whereas in (**b**) it is the minimum value of BS/BV for that VOI-1 (6.6 mm^–1^) that is only obtained when the VOI-1 is fully occupied by compact bone.

Unfilled and Pluronic F127 filled samples showed increased values of repaired bone volume (BV, Fig 6a) at 15 days after implantation, which were statistically significant compared with DBM-PF127 composite filled defect (*p* = 0.0004 *vs*. unfilled and *p* = 0.003 *vs*. Pluronic F127 filled) in VOI-1. Higher values of BV were found for all samples at 30 days when comparing to 15 day time point, while at 90 days all samples showed that almost 100% of the VOI-1 was filled by bone tissue, indicating a complete repair.

The porosity of the new bone in the VOI-1 was evaluated by mean of the surface-to-volume ratio (BS/BV) (Fig 6b). The DBM-PF127 composite filled defect showed a significant (p = 0.02) higher porosity(i.e., less compactness) compared to Pluronic F127 and control at 15 days after implantation, while no significantdifferences were observed among all groups at 30 and 90 days after implantation.

**Fig 7:**
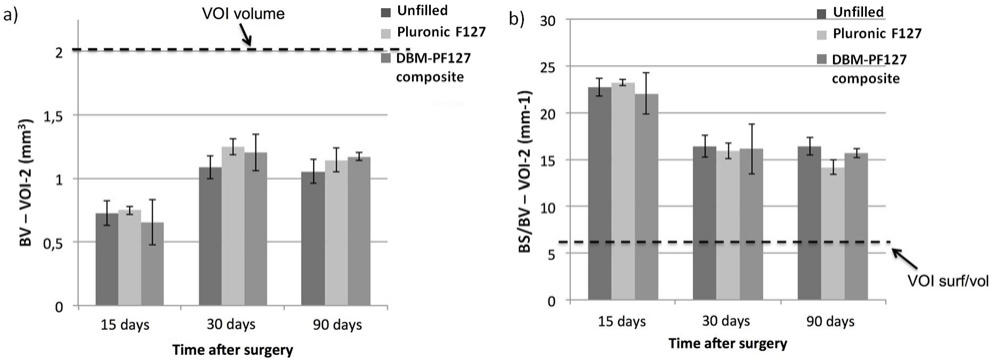
Quantitative results of bone repair as obtained with ex vivo micro-CT in VOI-2. In (**a**), the dashed line represents the total volume in the VOI-2 (2 mm^3^) which is the limit for 100% repair, whereas in (**b**) it is the minimum value of BS/BV for that VOI-2 (6.6 mm^–1^) that is only obtained when the VOI-2 is fully occupied by compact bone.

No significant differences were observed for the VOI-2 (endosteal, Fig 7a and 7b), where all groups showed comparable behaviors at the three time points, thus indicating that the repair of bone in the case of DBM-PF127 composite was prevalently shifted towards the medullary canal.

**Fig 8:**
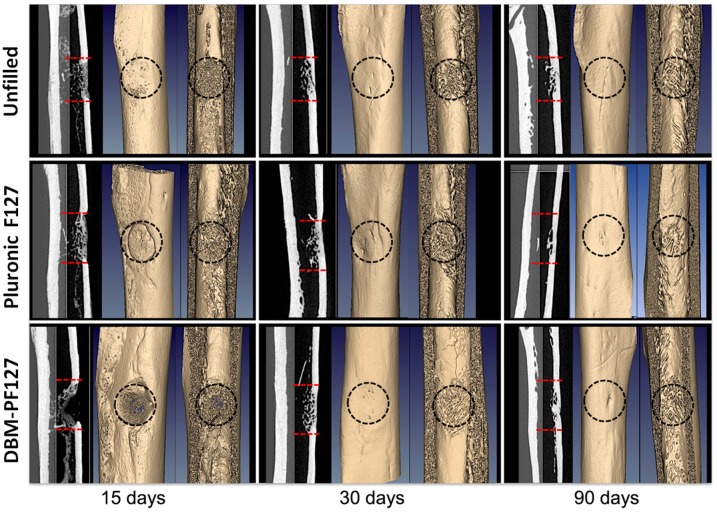
Micro-CT ex vivo images of rat femurs showing qualitatively the impact of the different treatments at the three time points. Dashed line indicate the defect edges.

**Fig 9:**
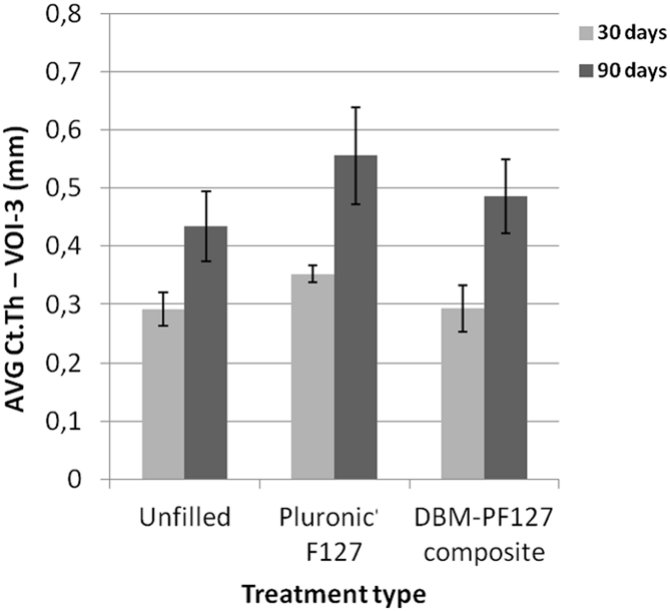
Quantitative results of bone thickness as obtained with ex vivo micro-CT in VOI-3.

Fig 8 shows qualitatively the impact of the different treatments at the three time points. At 15 days after implantation the control femurs appear almost completely repaired with a dense network of trabeculae in the endosteal face, as opposed with the femur treated with DBM-PF127 composite where compact bone is not yet observable. The sample treated with Pluronic F127 showed a partial formation of the cortical layer, plus a dense network of trabeculae in the endosteal face. At 30 days after implantation, all samples showed a complete closure of the lesion with formation of cortical bone. Samples treated with DBM-PF127 composite were remarkably more healed as compared to what observed after 15 days. The density of trabeculae in the endosteal face is comparable to that observable in the control. At 90 days after implantation, all samples showed complete healing with formation of compact bone in the cortical layer with good resorption of the trabecular component in the endosteal layer (as confirmed by the reduced BS/BV in VOI-2 for this time point, shown in Fig 7b). The progress of the resorption of the endosteal trabeculae can be even more appreciated by observing the differences in the Ct.Th at the center of the lesion between the groups at 30 days and 90 days (Fig 9). From this Figure it can be seen that the average cortical thickness in VOI-3 is significantly increased for all treatment types at 90 days vs. 30 days post-surgery (p = 0.03 for unfilled femurs, p = 0.02 for Pluronic F127, and p = 0.03 for DBM-PF127 composite). No significant differences of Ct.Th are observed between treatment types at the two time points under consideration (30 days and 90 days), even though the unfilled defects resulted thinner (Ct.Th = 0.435 ± 0.06 mm) as compared to those treated with Pluronic F127 (Ct.Th = 0.556 ± 0.083 mm, p = 0.27) and DBM-PF127 (Ct.Th = 0.486 ± 0.063 mm, p = 0.61). The femurs at 15 days have been excluded for the cortical analysis as the bone callus was still woven and immature to allow a meaningful assessment of this metrics.

### Histological results

**Fig 10:**
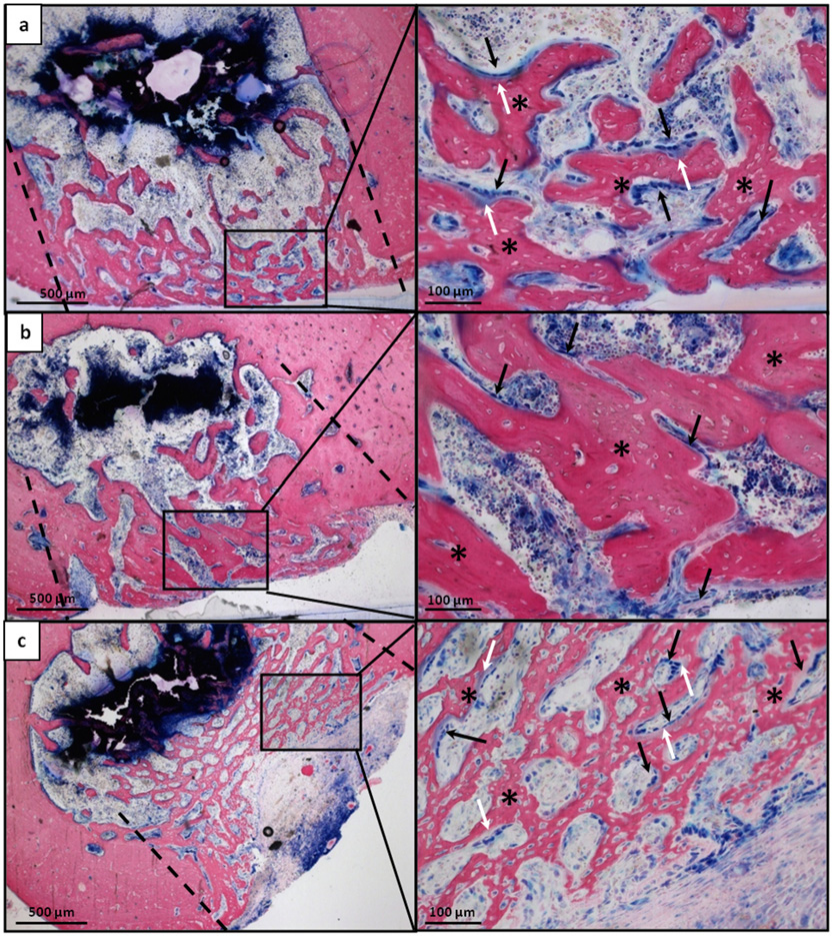
Histological microphotographs of femoral sections at the lesion site 15 days after implantation. **a**) Unfilled defect; **b**) Pluronic F127 filled defects; **c**) DBM-PF127 composite filled defects. Sections were stained with Stevenel’s/Van Gieson stain. In the left column the entire transversal section passing through the lesion is shown, in the right column high magnification of a portion of right column images is shown. Dashed line indicate the defect edges, (*) indicate the newly formed bone, Black arrow indicate osteoblast cells and white arrow indicate osteoid.

At 15 days unfilled defects and defects filled with Pluronic F127 showed consistent amount of new bone deposition founded mainly in correspondence of the original bone cortical layer, although few trabeculae were present in bone marrow central space (Fig 10a and 10b left column). At the first impression, there were no huge differences in the morphology of the defects filled with DBM-PF127 composite, albeit the consistency of the new bone deposition resembled a more internal localization due to the presence of the DBM-PF127 composite residual that acted as a scaffold capable of supporting bone regeneration in the hole left by the lesion. The new bone formation appeared thinner but more regular in its deposition, showing no interruption of the new bone with the native one’s at the original defect’s margins (Fig 10c left column). High-ranking of osteoid deposition and a great deal of osteoblasts were present at the external surface of the new deposited bone (Fig 10c, right column), similarly to unfilled defects (Fig 10a, right column) but differently from Pluronic F127 filled defects (Fig 10b, right column).

In the DBM-PF127 composite samples, the new trabeculae were distributed to fill the surface of the lesion as dense well-connected network with a regular, though thinner, shape. In the Pluronic F127 defects the trabeculae were organized as an irregular network and larger in their thickness. Finally, the unfilled samples showed a larger presence of not connected trabeculae but the thickness was similar to DBM-PF127 composite samples.

**Fig 11:**
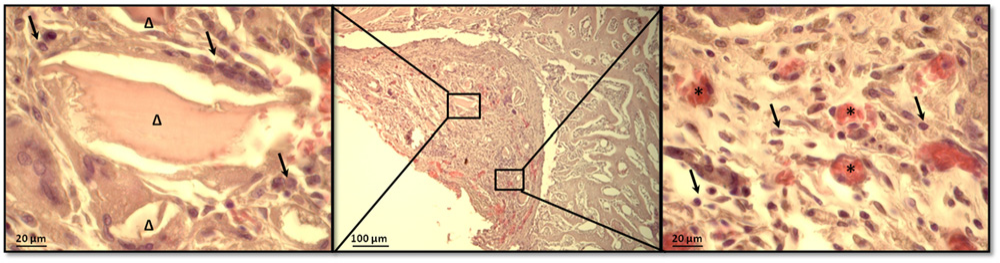
High magnification microscope observations of the femoral lesion filled with DBM-putty after 15 days of implantation. Centre: image at low magnification of DBM-PF127 composite residual occupying part of the defect in which wound healing process is taken over; Right: residual of DBM particles (Δ) at higher magnification; Left: presence of blood vessels (*). Black arrow indicate osteoblast like cells. Section were stained with Hematoxylin/eosin stain after paraffin embedded tissue protocol.

New bone deposition together with medullary cells infiltration and new blood vessels formation were observed in the DBM-PF127 composite filled defect also in paraffin embedded samples (Fig 11) indicating a positive ratio of the bone healing process.

**Fig 12:**
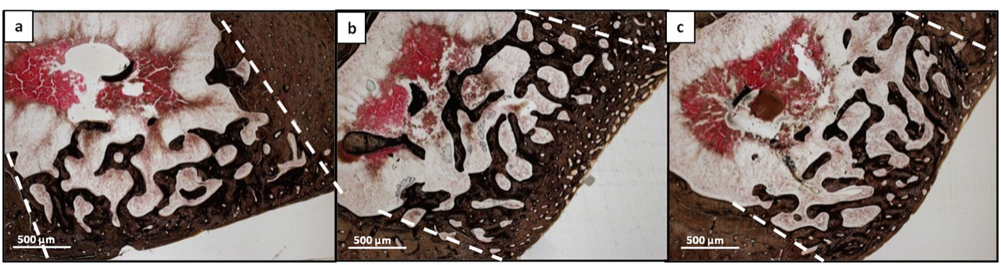
Histological microphotographs of femoral sections at the lesion site 30 days after implantation. **a**) Unfilled defect; **b**) Pluronic F127 filled defects; **c**) DBM-PF127 composite filled defects. Sections were stained with Von Kossa stain. Dashed line indicate the defect edges.

At 30 days after implantation all samples showed new bone remodeling as major event in the defect area noticeable as trabecular-like structures remained in the internal area, while at the external surface the bone acquired a more mature morphological feature, nevertheless, at this time point, a complete organization of the bone in lamellar deposition had not yet been reached by all samples (Fig 12). However, the histological analysis at day 30 after implantation evidenced a complete resorption of DBM-PF127 composite in the treated sample.

**Fig 13:**
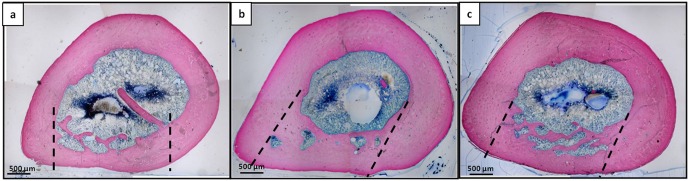
Histological microphotographs of entire transversal femoral sections passing through the lesion at 90 days after implantation. a) Unfilled defect; b) Pluronic F127 filled defects; c) DBM-PF127 composite filled defects. Sections were stained with Stevenel’s/Van Gieson stain. Dashed line indicate the defect edges.

The histological analysis at 90 days after implantation illustrated a complete closure of the lesions and a well integration of the new bone at the level of the edges of the defect in all samples (Fig 13) DBM-PF127 composite treated samples showed the regular restore of the bone thickness, as well as Pluronic F127 samples, instead the unfilled defect showed a thinner cortical layer. At this time point the density of the newly formed bone deposition was comparable for all three samples. Moreover, inflammatory reactions were absent at each time-point in DBM-PF127 composite and Pluronic F127 filled defects.

## Discussion

The importance of remodeling properties of bone substitutes is due to the fact that treating bone voids with individual particles, such as DBM, calcium phosphate and hydroxyapatite, may lead to incomplete filling of the defect or granule dispersion and loss during surgery [33].

These limitations might be overcome by using a carrier for developing injectable composites based on granules of DBM or calcium phosphate particles [34, 35]. Currently, different injectable or moldable bone composites based on various carrier materials, both natural and synthetic, including glycerol, Poloxamers, fibrin, hyaluronic acid, gelatin, chitosan, acellular matrix and PLGA have been studied [6–12]. These allow moldability of the product, i.e. its ability to be shaped and then placed to fit a defect of irregular geometry, as is encountered during surgery, and to be in direct contact with the bone surrounding the defect.

In recent years Pluronic F127, a well known thermoreversible biocompatible gel [15], has been largely studied as a carrier for drug administration in different medical applications [36, 37]. It has attracted particular interest for the development of a composite based on dissolving gels for delivery of biphasic calcium phosphate (BCP) and mesenchymal stem cells [38, 39]. Finally, Pluronic was used successfully for DBM delivery in rabbit calvaria model providing an almost complete bone regeneration at 90 days [40].

Gamma irradiation is frequently used to sterilize implanted devices. In spite of this, the effects of gamma irradiation on many polymers still remain to be studied. Concerning Pluronic F127 hydrogel it has been demonstrated that gamma irradiation at a low dose (40 Gy) did not have any significant effect [41]. However, higher gamma radiation doses, such as 25 kGy, the minimum required to achieve sterility assurance level, have been not investigated yet.

Regarding the DBM, cold gamma irradiation (-40 to -70°C) has been used to reduce the deleterious effects of irradiation, but other studies have shown that the low temperature alone could not prevent tissues from matrix damage and loss of osteoinductivity in DBM [42, 27]. Another study indicated that DBM could retain their osteoinductivity when it is in dry configuration and irradiated at low temperature (-40 to -70°C) [43].

In this study we have initially evaluated the effect produced by gamma irradiation on the physical-chemical properties of Pluronic and on BMPs amount released from irradiated and not irradiated DBM samples, respectively. All samples were stored in nitrogen atmosphere and gamma irradiated with a dose of 25 kGy at -80°C.

The evaluation of the physical and chemical characteristics of a 20% (w/v) Pluronic F127 solution was obtained comparing the rheological and infrared spectroscopy analysis of irradiated *vs* not irradiated solutions. In particular, no change in sol-gel transition temperature (25°C) and no effect on the principal functional groups in the IR spectra were detected. However, the increase of G’ value at temperature > 30°C of gamma irradiated Pluronic F127 could be probably due to crosslinking effect as previously observed by Ibrahim et al. [28]. Furthermore, the derivative analysis of the spectra collected for irradiated Pluronic F127 did not show any band at 1727 cm^-1^ (carbonyl group). The appearance of a carbonyl band indicates a degradation effect induced by gamma irradiation at 25 kGy dose [44].

We have shown instead that the gamma irradiation of DBM, in the same conditions described for Pluronic F127 solution, caused a 20% decrease in BMP-2 and BMP-7 concentration compared to the initial values. In this regard, Han and colleagues demonstrated that the sterilization by gamma radiation can determine a decrease of osteoinductive capacity by DBM-based products, mainly caused by factors such as temperature of sterilization, radiation dose and atmosphere of conservation and they found that dry condition at 25 kGy are better respect to hydrated conditions [26].

After the above described observations, an injectable DBM-PF127 composite, containing 40% of DBM (w/w), was prepared and sterilized by gamma irradiation at 25 kGy, -80°C, and in nitrogen atmosphere, as well as the Pluronic F127 and the DBM taken separately. Samples were tested as a filler of femoral defects (diameter 3 mm) in a Wistar rat model. A comparison was made with Pluronic F127 filled and unfilled (control) defects. Unfilled defect was chosen as control since it has been reported that a young (approx. 3 months) healthy rat, such as those used in this study, has high remodeling capability in terms of bone formation [45]. Therefore this animal model can be reasonably assumed optimal to evaluate bone regeneration.

Samples of not gamma irradiated DBM-PF127 composite were not implanted to avoid undesirable infections that could affect the experiment; and to reduce the use of animals according to the 3Rs of ethical animal use: replacement, reduction and refinement [46].

The quantitative Micro-CT analysis has been used effectively by other authors to evaluate the healing of rat femoral segmental defect in term of bone volume and tissue mineral density concerning the use of an absorbable collagen sponges carrying BMP-2 [47] and individual DBM particles [33]. Destructive mechanical testing is also used in some cases to assess directly the mechanical performance of the healed bone; in this study, this type of testing was not possible because it is incompatible with histological analysis and hence it would have required to increase the number of sacrificed animals. It is known that there is a direct relationship between bone stiffness and bone volume fraction in the femur diaphysis [48], so we believe that increasing of the group size or the number of groups to include mechanical testing would violate the 3 R’s law as it does not add independent information to that already available from micro-CT imaging.

In our study the micro-CT analysis in cortical region (VOI-1) at 15 days of bone lesions filled with DBM-PF127 composite evidenced a minor formation of bone trabeculae respect to unfilled and Pluronic F127 filled defects, while a dense trabecular network and thick cortical region was observed at 30 and 90 days in all analyzed samples. At day 90 a complete tissue repair was observed in all samples, indicating a complete resorption and integration of DBM-PF127 composite in the wounded area replaced by newly formed bone tissue.

For the absence of trabeculae formation in cortical region in the composite filled defect at 15 days it can be hypothesized that when DBM-PF127 composite was implanted, Pluronic F127 started to dissolve in biological fluids [49], during this process, the space available among DBM granules may have increased over time, allowing soft tissues (including capillaries) to gradually enter the implants with following recruitment of medullary cells and osteoblast-like cells, finally resulting in bone formation. A similar behavior related to polymeric gels incorporating biphasic calcium phosphate particles was observed by Barbieri et colleagues [38]. Furthermore, the absence of significant improvement in shortening the healing process in the DBM-PF127 composite filled defect respect unfilled and Pluronic F127 filled defect at 90 days can be caused by slower dissolution rate of the Pluronic F127 in the core respect to outer external layer of injected composite according to finding of Barbieri et al. [38] and by a decrease in degradation rate of the DBM particles as previously described by Araújo et al. from 21 days to 4 months [33].

The results of the micro-CT analysis in medullary canal (VOI-2) showed comparable behavior for all the three groups at each time-point indicating that the bone reparation in the case of DBM-PF127 composite was prevalently shifted towards the medullary region.

The histological analysis at 15 days after implantation confirmed the results obtained by micro-CT. DBM-PF127 composite filled defects showed a lower amount of new bone deposition in cortical region respect to the other samples, however the trabeculae appear to have a regular thickness and an interconnected network. H&E staining showed that DBM-PF127 composite residuals were localized at the injection site and a high number of osteoblast-like cells and capillaries were present around the DBM particles in the cortical region of the new bone. DBM particles were not observed in the medullar cavity demonstrating that the carrier is able to maintain the particles *in situ*. In previous studies, DBM particles applied without carrier in rat femur were found at 16 weeks in the medullary canal [33], while the use of Pluronic F127 combined with calcium phosphates enhanced particles handling and moldability without any negative effects on biocompatibility [50]. Finally, there was no evidence of an inflammatory response to Pluronic F127 materials, similar findings were observed in sheep intramuscular implantation of Pluronic F127 used as carrier for the delivery of biphasic calcium phosphate [38] and in rabbit parietal bones of bone substitute containing Pluronic as excipient [40].

At day 30 after implantation no residual DBM deposits were observed, thus evidencing the complete resorption of DBM-putty. Moreover, all defects showed new bone trabeculae remodeling as major event, the cortical bone was healed and no differences were noticeable among samples. The histological analysis at 90 days after implantation demonstrated a complete remodeling of cortical bone without visible margins of cortical defects in all samples. Moreover in the DBM-PF127 composite treated defects no bony islands were present in the medullary cavity. A higher cortical bone thickness was found in DBM-PF127 composite and Pluronic F127 treated defects with respect to the unfilled lesions. Finally, the new bone tissue presents all the features of mature bone tissue, evidenced by the presence of osteocytes without significant difference in bone density compared to the control.

Previously study demonstrated that PLGA or collagen combined with bone marrow or tricalcium phosphate respectively may retard the healing process at bone marrow level in rat tibial marrow respect to DBM, in fact the DBM particles are reabsorbed with the same rate of the remodeling process of the trabecular bone [51].

## Conclusions

The present study showed that a gamma irradiated DBM-PF127 composite, although it may have undergone a reduction of about 20% in the concentration of BMP-2 and BMP-7 (as shown by the exposure of DBM to gamma irradiation), maintains good bone regeneration capability. Samples applied to a lesion affecting bone medullary and cortical regions in a healthy, young Wistar rat model shown comparable results in term of density and thickness of the new formed tissue, and similar cellular components and matrix organization, respect to unfilled defect caused in the medial femoral diaphysis. This is demonstrated by the presence of progenitor cells and osteoblast like cells at 15 days of implantation, and by complete resorption of the scaffold with regeneration of cortical tissue damaged from 30 to 90 days of implantation. Furthermore, the DBM-PF127 composite showed an excellent biocompatibility with no presence of inflammatory cells in the peri-implantation region.

In conclusion, the use of gamma irradiation in sterilization of DBM-PF127 composite should be used under precise controlled condition as irradiation can affect both physical and chemical properties of components, especially at high irradiation doses.

A DBM-PF127 composite containing 40% (w/w) of DBM and 20% (w/v) of Pluronic F127 could be a sterile product with acceptable properties of bone regeneration after gamma irradiation with a dose of 25 kGy at -80°C in nitrogen atmosphere. This formulation may represent a convenient scaffold to fill the defects and promote natural regeneration of damaged bone tissue in pathological conditions, such as tumors and cysts, or traumatic bone losses.
